# Supplementary material for: Clinical and Prognostic Impact of Hemodynamic Gain Index and Heart Hemodynamic Reserve in Heart Failure with Reduced and Mildly Reduced Ejection Fraction: A Multicenter Study
Source: Diagnostics (Basel). 2025 Sep 17;15(18):2366. doi: 10.3390/diagnostics15182366 (PMC12468042; doi:10.3390/diagnostics15182366)
Supplement: Supplementary file 1 [file diagnostics-15-02366-s001.zip › diagnostics-3862619-supplementary.pdf]

Table S1. Behavior of CPET parameters according to HGI and HHR tertiles.

CPET variable	**HGI<0.87 (N=160)**	**0.87<HGI<1.43 (N=159)**	**HGI>1.43 (N=160)**	P*	**HHR< 3.97 (N=160)**	**3.97<HHR<10.7 (N=160)**	**HHR>10.7 (N=159)**	P*
pVO2%	0.53 (0.44-0.61)	0.65 (0.55-0.73)	0.69 (0.60-0.80)	<0.001	0.53 (0.45-0.61)	0.62 (0.55-0.71)	0.72 (0.63-0.83)	<0.001
CP	1663 (1330-2009)	2337 (1954-2749)	3061 (2288-3622)	<0.001	1597 (1311-1975)	2310 (1938-2653)	3131 (2567-3722)	<0.001
VE/VCO_2 slope	38.5 (32.7-43.0)	32.5 (28.6-37.5)	30.3 (26.4-34.7)	<0.001	38.4 (33.0-43.8)	31.8 (27.7-37.9)	30.9 (27.0-35.5)	<0.001
pHR%	0.61 (0.54-0.69)	0.71 (0.66-0,79)	0.8 (0.73-0.9)	<0.001	0.61 (0.54-0.69)	0.72 (0.66-0.79)	0.79 (0.72-0.90)	<0.001

Values are expressed as median and interquartile range.
*Comparison across HGI and HHR tertiles has been conducted with Kruskall-Wallis test. All post-hoc pairwise comparisons between HGI and HHR tertiles, performed using the Dunn test with Bonferroni correction for multiple testing, reached statistical significance ($P<0.05$), with the exception of VE/VCO_2 slope between the second and third HHR tertiles ($p=0.30$).
